# Effects of different treatments on the prognosis of patients with single-organ oligometastasis of esophageal cancer after surgery–a retrospective single center study

**DOI:** 10.3389/fonc.2025.1504410

**Published:** 2025-03-06

**Authors:** Jinrui Xu, Chunyang Song, Jingyuan Wen, Wenzhao Deng, Xuan Wang, Shuguang Li, Jingwei Su, Wenbin Shen

**Affiliations:** Department of Radiation Oncology, The Fourth Hospital of Hebei Medical University, Hebei Clinical Research Center for Radiation Oncology, Shijiazhuang, China

**Keywords:** esophageal tumor/esophageal cancer, surgical treatment, single organ oligometastasis, radiotherapy and chemotherapy, prognosis, nomogram

## Abstract

**Objective:**

To investigate the impact of clinicopathological factors on the prognosis of patients with single-organ oligometastasis of esophageal cancer (soOMEC) following surgery and to develop prognostic nomograms.

**Methods:**

This was a retrospective analysis of 144 patients with soOMEC after surgery in a single center who met the inclusion criteria. First, Cox univariate and multivariate models were used to analyze (SPSS 25.0 statistical software) the characteristics of patients, and independent prognostic factors for postoperative overall survival (OS) and OS after oligometastasis (OM-OS) were determined. Prognosis was analyzed using R language software, nomograms were created based on the Cox multivariate analysis results, a bootstrap method (b=200) was used for internal validation, and receiver operating characteristic (ROC) and calibration curves were used to validate the models.

**Results:**

From January 2014 to December 2017, a total of 1595 patients with esophageal cancer received R0 resection. As of the end of the follow-up period, 144 patients had single-organ oligometastasis (soOM). The median time to oligometastasis (TTO) in the whole group of patients was 14.2 months, and the 1-, 3-, and 5-year OS rates were 75.7%, 28.2%, and 13.3%, respectively. The median OS was 25.0 months (95% confidence interval (CI): 21.8-28.2); the 1-, 2-, and 3-year OS rates after distant metastasis (DM-OS) were 25.5%, 13.3%, and 7.2%, respectively, and the median DM-OS was 5.5 months (95% CI: 3.9-7.1). The Cox multivariate analysis results showed that three indicators, i.e., TNM stage (hazard ratio (HR)=2.192, 95% CI: 1.441-3.336, P=0.000), TTO (HR=0.119, 95% CI: 0.073-0.194, P=0.000), and treatment after DM (HR=0.784, 95% CI: 0.970-0.025, P=0.025) were independent prognostic factors affecting the OS of patients; TTO (HR=0.669, 95% CI: 0.455-0.984, P=0.041) and treatment after DM (HR=0.713, 95% CI: 0.559-0.910, P=0.007) were independent prognostic factors affecting the DM-OS of patients. Using the Cox multivariate analysis results, prediction nomograms for total OS and DM-OS of patients were established. In the validation of the nomogram models, the areas under the curve (AUCs) for the 1-, 3-, and 5-year total OS were 0.930, 0.927, and 0.928 in the training set and 0.705, 0.856, and 1 in the validation set, respectively; the AUCs for the 1-, 2-, and 3-year DM-OS were 0.904, 0.923, and 0.908 in the training set and 0.928, 0.842, and 0.895 in the validation set, respectively. The results showed that the two models have strong discriminative ability and good clinical promotion and application value.

**Conclusions:**

Aggressive local therapy combined with systemic chemotherapy can benefit patients with soOMEC after surgery, and for patients with OM appearing at 1 year after surgery, aggressive radiotherapy or combined chemotherapy is expected to improve the prognosis and prolong OS. The nomogram models developed in this study demonstrated strong predictive performance in internal validation and hold potential as clinical tools for estimating the prognosis of patients and assisting in treatment decision-making. However, their true clinical utility and generalizability require further validation through larger, multicenter, and prospective studies.

## Introduction

Esophageal cancer is one of the most common malignant tumors in the digestive tract, with high morbidity and mortality (1). Recent studies have shown that patients with esophageal cancer who receive neoadjuvant chemoradiotherapy combined with surgery have a better prognosis, and 10-year survival results have been reported (2); however, overall, 20% to 50% of patients with esophageal cancer develop distant metastases (DMs) during posttreatment follow-up (3–5). Importantly, there are currently no unified strategies or effective treatments for patients with postoperative recurrence or metastasis (6, 7). In recent years, combined immunotherapy has shown good efficacy in the treatment of patients with advanced esophageal cancer (8); however, there is currently a lack of relatively complete evidence-based medical evidence for patients with postoperative metastasis. Patients with esophageal cancer who experience postoperative DMs may have a worse prognosis than those with postoperative locoregional recurrence (9). Compared with the available treatments for patients with postoperative locoregional recurrence, there may be fewer available treatments for patients with DMs. Studies in recent years have shown that patients with oligometastasis (OM) may be a relatively independent population of patients with DMs (10, 11). For patients with OM, primary lesions can be stably controlled, lesions are in a transitional stage between limited and extensive metastases, the number of metastases is small, and metastases are located in specific tissues or organs; therefore, the tumor load is limited, and these metastases can be treated with existing therapeutic interventions, thus improving the survival of some patients (12, 13). There have been many studies on oligometastatic patients with non-small cell lung cancer, and relevant reports have shown positive results (14). There are few studies on treatments for oligometastatic patients with esophageal cancer after surgery. To further clarify the prognosis of oligometastatic patients with esophageal cancer after surgery, for this study, only patients with soOMEC after surgery were selected, and herein, were report the results of a retrospective analysis of 144 patients with soOMEC after surgery in a single center.

## Materials and methods

The work has been reported in line with the STROCSS criteria. Inclusion and exclusion criteria: The inclusion criteria were as follows: patients who underwent radical resection of esophageal cancer in our hospital and postoperative pathology confirmed esophageal malignancy; patients who did not receive neoadjuvant therapy before surgery(This criterion was established to reduce variability and potential confounding introduced by neoadjuvant treatment, which can significantly impact tumor biology, treatment response, and long-term prognosis. By focusing on patients treated with surgery as the primary intervention, we aimed to ensure a more homogeneous study population for analyzing postoperative prognostic factors); patients who attended regular re-examinations at our hospital after surgery; patients with distant organ metastasis confirmed by magnetic resonance imaging (MRI) or positron emission tomography (PET)/computed tomography (CT) who could not undergo surgical treatment after a surgical evaluation and in whom metastasis occurred in a single organ, with ≤5 metastases; patients with well controlled primary lesions and no regional lymph node recurrence; patient with an expected survival after metastasis ≥ 3 months; patients with a Karnofsky performance scale (KPS) score ≥ 70 after metastasis; patients with at least one measurable lesion; and patients with complete follow-up and clinical data.

Patients with other malignancies or diseases in important organs, such as the heart, liver, kidney, or blood system, were excluded, and patients with local regional recurrence in the thoracic cavity and only lymph node metastasis or DMs at different times were excluded. This study was conducted in accordance with the principles of the 1975 *Declaration of Helsinki* and its subsequent amendments or similar ethical standards and was approved by the Ethics Committee of the Fourth Hospital of Hebei Medical University. Because this was a retrospective study, the Ethics Committee of the Fourth Hospital of Hebei Medical University waived the requirement for informed consent.

### General clinical data

From January 2014 to December 2017, a total of 144 eligible patients (120 males and 24 females) were identified. The calculated age from the date of surgery ranged from 40 to 77 years, with a median age of 60 years. Eighty patients had a smoking history, 44 had a drinking history, and 13 had a family history; 117 patients had an Eastern Cooperative Oncology Group (ECOG) score of 0 at the time of surgery, and 73 patients had an ECOG score of 0 after DM. There were 18, 93, and 33 patients with esophageal lesions in upper, middle and lower thoracic segments, respectively. The length of esophageal lesions before surgery, as determined using X-ray angiography, ranged from 1.5 to 10.0 cm, with a median of 5.0 cm. Postoperative pathology confirmed 124 cases of squamous cell carcinoma, 18 cases of adenocarcinoma, and 2 cases of small cell carcinoma. However, the primary focus of this study was on patients with squamous cell carcinoma (SCC), given its predominant representation in the cohort. Patients with adenocarcinoma and small cell carcinoma were included for initial analysis to ensure comprehensive data collection but were subsequently excluded from subgroup and detailed analyses due to potential heterogeneity. Using the 8th edition of the American Joint Committee on Cancer (AJCC) tumor‐node‐metastasis (TNM) staging system for esophageal cancer, there were 6, 19, 125, and 8 patients with postoperative pathological stage T1, T2, T3, and T4 disease, respectively; there were 56, 52, 29, and 7 patients with postoperative pathological stage N0, N1, N2 and N3 disease, respectively; and there were 5, 46, 16, 42, 19 and 16 patients with TNM stages I, IIA, IIB, IIIA, IIIB and IIIC disease, respectively. A total of 49, 32, and 22 patients received adjuvant postoperative chemotherapy (POCT), postoperative radiotherapy (PORT), and postoperative chemoradiotherapy (POCRT) respectively, and the remaining 41 patients did not receive any form of postoperative adjuvant treatment.

### Diagnosis of DM

DM was defined as haematogenous metastasis to parenchymal organs, bone, thoracic peritoneum, pericardium, and chest and abdominal walls, excluding lymph node metastasis outside the resection site. Single-organ metastases were defined as metastases confined to a single tissue or organ. OM was defined as ≤5 metastases in a single organ. The diagnosis of metastases was mainly based on histopathology or diagnostic imaging results. All patients underwent ultrasound of the superficial lymph nodes, head MRI, CT and/or MRI scans of the chest and abdomen, and whole-body emission CT (ECT). Thirty-nine patients underwent PET/CT; 16 patients with lung metastases and 7 patients with liver metastases underwent CT or ultrasound-guided aspiration biopsy and pathological diagnosis; 5 patients with subcutaneous metastases and 6 patients with chest wall metastases underwent needle aspiration biopsy and pathological diagnosis; and patients with metastases in the pleural cavity and the pericardial cavity underwent exfoliative cytology of effusions and pathological diagnosis.

### Treatment after DM

Most of the patients received systemic chemotherapy (71 patients, 49.3%), 29 patients (20.1%) received involved-field radiation therapy (IFRT), 21 patients (14.6%) received combined chemoradiotherapy, and 23 patients (16.0%) received optimal maintenance therapy only. The chemotherapy regimens were mainly platinum-based combination chemotherapy; for 79 patients, the combination therapy included paclitaxel (PTX)-based regimens, and for 13 patients, the combination therapy included 5 fluorouracil-based regimens. Radiotherapy was performed with intensity-modulated conformal radiotherapy. For external irradiation, a 6 MV X-ray linear accelerator was used. The linear quadratic model was α/β=10 Gy, and the median biologically effective dose (BED_10_) was 50 Gy (range: 30-70 Gy).

### Follow-up

The main methods of follow-up were telephone calls and outpatient re-examinations. Re-examinations occurred once every 1-6 months in the first year, once every 3-6 months in the second year, and once every 6-12 months in the following years. The follow-up deadline was December 31, 2021.

### Statistical analysis

SPSS 25.0 statistical software (IBM Corp., Armonk, NY, USA) was used for the data analyses. The Kaplan-Meier (KM) method was used to calculate the overall survival (OS) rate and the survival rate after OM (OM-OS). The log-rank test and univariate prognostic analysis were performed, and a Cox proportional hazards model was used for the multivariate analysis of total OS and OM-OS (Forward: LR method – forward stepwise regression method based on maximum likelihood estimation) to assess the independent prognostic factors for this group of patients. Variables with P<0.1 in the univariate analysis were included in the Cox multivariate regression model. Hazard ratios (HRs) with 95% confidence intervals (CIs) were calculated, and P<0.05 was considered statistically significant.

Based on the Cox multivariate analysis results, nomograms of total OS and OM-OS for this group of patients were established. First, using the univariate analysis results, covariates with P<0.05 were selected. Using R version 4.2.1 (R Foundation for Statistical Computing, Vienna, Austria), the dataset was randomly divided into a training set and a validation set, and statistical tests of differences between groups were performed. Among them, the t test or Wilcox rank sum test was used for continuous variables based on normality and homogeneity of variance; the chi-square test or Fisher’s exact test was used for categorical data (including binary and multiclassification data) based on normality and homogeneity of variance; and the log-rank test was used for survival data. There were 108 samples in the training set and 36 samples in the validation set. The R language rms and survival toolkits were used to perform Cox multivariate regression analysis of statistically significant covariates and draw nomograms based on the results. The obtained total OS and OM-OS models passed internal validation using the bootstrap method with 200 repeated samplings. To evaluate the model, receiver operating characteristic (ROC), calibration and decision curves were used. Time-dependent ROC curves were drawn using the R language timeROC toolkit, and the areas under the receiver operating characteristic curve (AUCs) were calculated to evaluate the discriminative ability of the nomograms. The accuracy of the nomograms was assessed using calibration curves. The ggDCA toolkit in R language was used to perform decision curve analysis (DCA) to quantify the net benefit for patients under different threshold probabilities to evaluate clinical utility.

## Results

### Patient selection analysis

From January 2014 to December 2017, a total of 1,595 patients with esophageal cancer received R0 surgical resection, and 670 (42.0%) patients experienced post-treatment failure by the date of patient death or the end date of follow-up, with 360 (22.6%) experiencing local recurrence alone, 148 (9.3%) experiencing local recurrence combined with DM, and 162 (10.2%) experiencing DM alone. Among the 162 patients with DM alone, 144 (88.9%) patients had single organ metastasis with ≤5 metastases; the other 18 (11.1%) patients had metastases in ≥2 organs or single-organ metastasis with >5 metastases.

### Analysis of single organ oligometastasis (soOM)

Among the 144 patients, 60 patients with pulmonary metastasis had unilateral lung metastasis, with 1 to 5 lung metastases (median, 3). among the 60, 12 received maintenance therapy, 32 received chemotherapy alone, 3 received radiotherapy alone, and 13 received combined chemoradiotherapy. There were 34 patients with liver metastases, with 1 to 4 metastases (median, 2); among them, 5 patients received maintenance therapy, 27 patients received systemic chemotherapy alone or combined with interventional chemotherapy, and 2 patients received combined chemoradiotherapy. There were 24 patients with bone metastases, mainly in the vertebral body, pelvis, rib and scapula; among them, 1 patient received maintenance therapy, 2 patients received chemotherapy alone, 17 patients received radiotherapy alone, and 4 patients received combined chemoradiotherapy. There were 6 patients with brain metastases, with 1-3 metastases (median, 1); among them, 1 received maintenance therapy, 4 received radiotherapy alone, and 1 received combined chemoradiotherapy. There were 5 patients with subcutaneous metastasis, with 1-3 metastases; among them, 4 patients received chemotherapy, and 1 received radiotherapy due to local symptoms caused by large metastases. There were 6 patients with chest wall metastasis: 4 with metastases in the left chest wall and 2 with metastases in the right chest wall; among them, 1 patient received chemotherapy alone, 4 patients received radiotherapy alone, and 1 patient received combined chemoradiotherapy. There were 8 patients with pleural cavity metastasis: 6 with unilateral pleural cavity metastasis and 2 with bilateral pleural cavity metastasis; among them, 3 patients received maintenance therapy and 5 received chemotherapy alone; 1 patient had pericardial metastasis and received maintenance therapy. The majority of the cohort (86.1%) consisted of patients with squamous cell carcinoma (SCC), which served as the primary focus of our analysis. Patients with adenocarcinoma and small cell carcinoma, though initially included for descriptive purposes, were excluded from subgroup and detailed analyses due to their small numbers and distinct biological characteristics. This decision aimed to reduce heterogeneity and ensure more reliable conclusions specific to SCC.

### Analysis of patient survival

The time of DM for all patients was 1.0-79.3 months, with a median of 14.2 months and an average of 20.0 months. The overall 1-, 3-, and 5-year OS rates for all patients were 75.7%, 28.2%, and 13.3%, respectively, and the median OS time was 25.0 months (95% CI: 21.8-28.2). The 1-, 2-, and 3-year OS rates after distant metastasis (DM-OS) were 25.5%, 13.3%, and 7.2%, respectively, and the median OS time was 5.5 months (95% CI: 3.9-7.1).

### Analysis of factors influencing patient prognosis

The univariate analysis results showed that six indicators, i.e., length on X-ray, pT stage, pN stage, TNM stage, time to oligometastasis (TTO) and treatment after DM, were prognostic factors affecting the OS of patients (X^2^ = 13.114, 4.430, 9.309, 17.700, 72.835, 9.938, P=0.000, 0.035, 0.010, 0.000, 0.000, 0.019) and that three indicators, i.e., TNM stage, TTO and treatment after DM, were prognostic factors affecting OM-OS (X^2^ = 4.909, 7.703, 12.807, P=0.027, 0.006, 0.005) (**Table 1**).

**Table 1:** The univariate analysis of factors influencing patient prognosis.

Characteristic	N	OS (%)		Median (M)	X^2^	P	OM-OS (%)		Median (M)	X^2^	P
		3-y	5-y				1-y	3-y			
Sex					0.078	0.780				0.299	0.584
Male	120	29.6	11.9	25.0			26.8	7.2	5.6		
Female	24	20.8	20.8	22.0			18.8	7.0	3.7		
Age					0.012	0.913				3.532	0.060
≤59	69	27.5	12.9	25.0			31.9	9.2	6.8		
>59	75	28.8	13.7	25.0			19.4	5.6	4.6		
Smoking					0.251	0.616				0.003	0.957
non-smoker	64	29.7	15.5	25.0			28.8	7.0	4.6		
smoker	80	26.9	11.5	22.0			22.8	7.6	5.6		
ECOG performance status					0.086	0.770				0.042	0.838
0	117	27.0	16.5	25.0			23.8	6.2	4.8		
1	27	42.9	0.0	30.0			14.3	0.0	5.6		
Location of primary tumor					1.079	0.583				5.145	0.076
Upper third	18	30.9	18.5	25.0			6.0	0.0	2.0		
Middle third	93	24.7	11.7	24.0			23.7	6.7	5.5		
Lower third	33	36.4	21.2	25.0			41.5	9.6	7.0		
Length on X-ray					13.114	0.000				3.305	0.069
≤5cm	51	45.1	31.4	36.0			34.3	14.1	6.8		
>5cm	93	18.7	5.5	21.0			20.7	3.8	4.6		
Pathology					3.819	0.051				1.645	0.200
squamous cell carcinoma	124	32.0	15.5	25.0			25.5	8.5	5.6		
non-squamous cell carcinoma	20	5.0	0.0	24.0			25.0	0.0	3.7		
Differentiation					0.053	0.818				0.657	0.418
non/low	28	25.0	17.9	24.0			24.5	8.2	6.8		
middle/high	116	28.9	14.0	25.0			25.8	6.7	5.0		
pT stage					4.430	0.035				1.843	0.175
pT1+pT2	31	48.4	25.4	30.0			31.5	10.7	6.8		
pT3+pT4	113	22.5	9.9	23.0			23.7	6.0	4.8		
pNstage					9.309	0.010				0.856	0.652
P N0	56	48.2	17.9	35.0			28.3	10.1	5.6		
pN1	52	13.5	11.5	21.0			23.1	7.7	4.6		
pN2 + 3	36	17.9	8.9	18.0			24.4	3.1	5.9		
TNM stage					17.700	0.000				4.909	0.027
I+II	67	46.3	22.1	33.0			30.5	14.1	6.7		
III	77	12.1	5.4	21.0			21.1	1.4	4.6		
Postoperative Adjuvant treatment					1.705	0.636				3.641	0.303
no	41	24.4	7.3	25.0			14.6	2.4	4.6		
POCT	49	30.6	18.4	22.0			31.8	9.6	6.0		
PORT	32	25.0	12.5	25.0			28.1	7.5	5.0		
POCRT	22	34.3	14.7	26.0			28.7	9.6	3.5		
Time to oligometastasis (TTO)					72.835	0.000				7.703	0.006
≤12month	60	6.7	3.3	11.0			16.7	1.7	4.6		
>12month	84	43.5	23.0	33.0			31.9	11.8	6.8		
Treatment after DM					9.938	0.019				12.807	0.005
Maintenance therapy	23	21.7	13.0	25.0			8.7	0.0	3.6		
CT	71	19.7	5.6	22.0			21.1	1.7	4.8		
RT	29	40.3	14.7	30.9			39.0	4.7	5.9		
CRT	21	47.6	38.1	31.0			41.6	36.4	8.0		
The site of metastasis					4.264	0.234				4.985	0.173
lung	60	30.0	20.0	26.0			24.7	10.5	4.6		
liver	34	23.5	5.9	17.0			12.7	0.0	3.5		
bone	24	40.4	17.9	30.0			26.1	8.7	5.6		
others	26	19.2	7.7	21.0			42.3	7.7	8.0		

**Figure 1:**
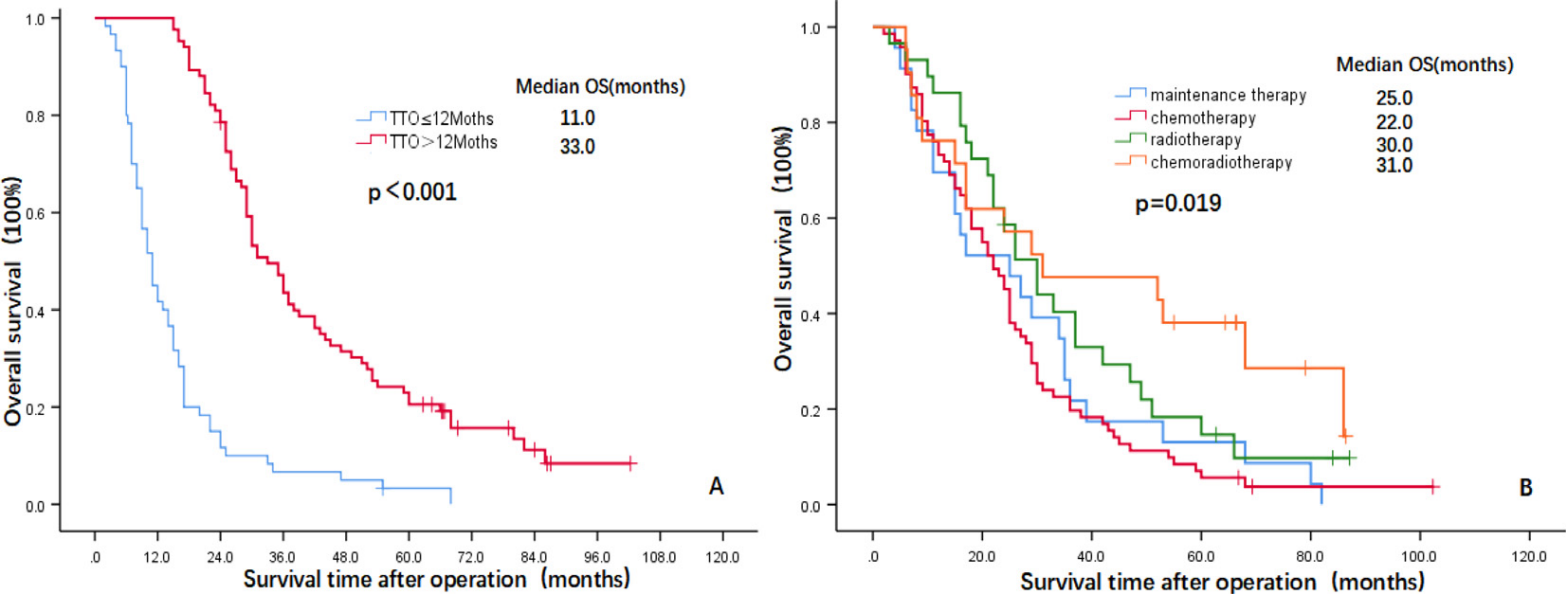
Kaplan–Meier curves showing overall survival (**A**: TTO, **B**: Treatment after DM). OS, overall survival; TTO, the median time to oligometastasis; DM, distant metastasis.

**Figure 2:**
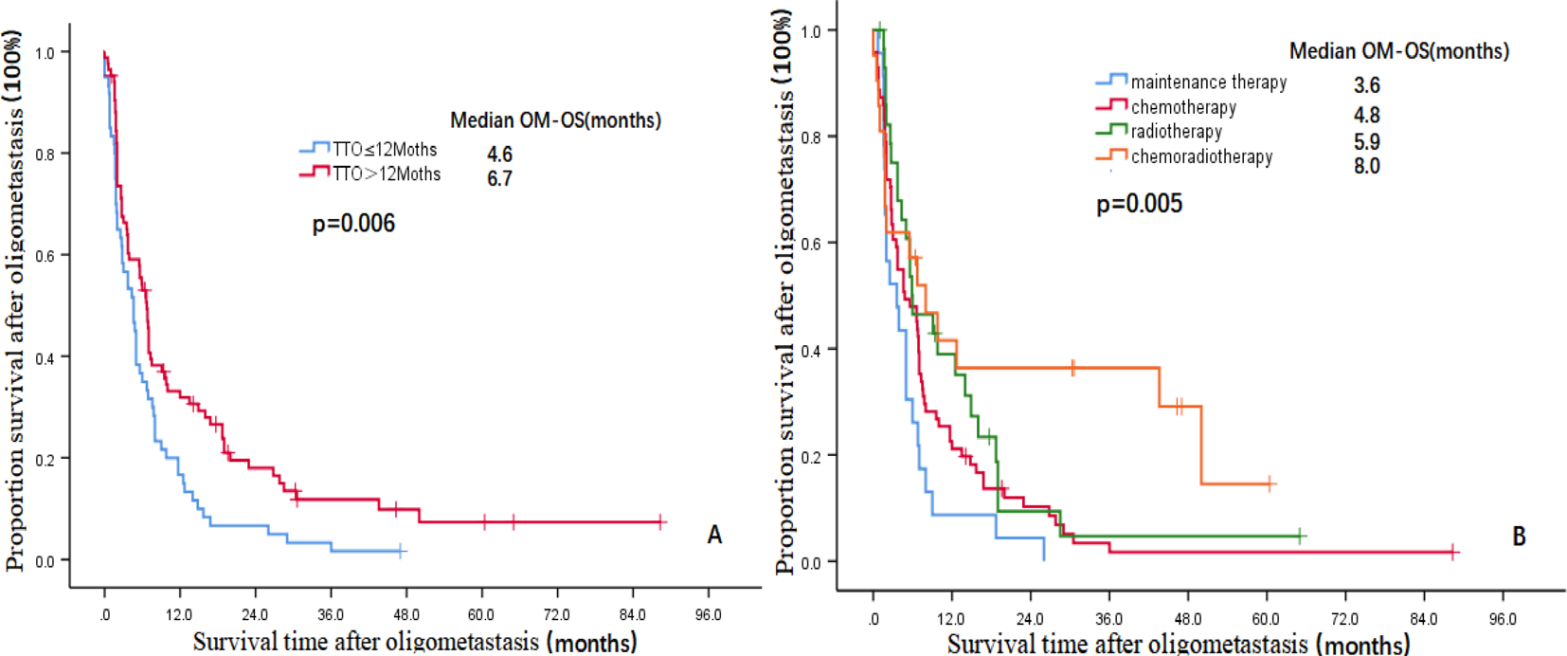
Kaplan–Meier curves showing the survival rate after OM (**A**:TTO, **B**:Treatment after DM)OM-OS, overall survival after oligometastasis; TTO, the median time to oligometastasis; DM, distant metastasis.

The multivariate analysis results showed that three indicators, i.e., TNM stage (HR=2.192, 95% CI: 1.441-3.336, P=0.000), TTO (HR=0.119, 95% CI: 0.073-0.194, P=0.000) and treatment after DM (HR=0.784, 95% CI: 0.970-0.025, P=0.025) were independent prognostic factors affecting the OS of patients and that two indicators, i.e., TTO (HR=0.669, 95% CI: 0.455-0.984, P=0.041) and the treatment after DM (HR=0.713, 95% CI: 0.559-0.910, P=0.007), were independent prognostic factors affecting OM-OS (**Table 2**). **Figures 1**, **2** show the effects of TTO and treatment after DM on total OS and OM-OS.

**Table 2:** The multivariate analysis of factors influencing patient prognosis.

Variable	B	S.E.	χ^2^	P	HR	95%CI	
						lower limit	upper limit
Total OS							
pTNM stage	0.785	0.214	13.434	0.000	2.192	1.441	3.336
TTO	-2.130	0.249	73.086	0.000	0.119	0.073	0.194
Treatment after DM	-0.244	0.109	5.016	0.025	0.784	0.633	0.970
OM-OS							
TTO	-0.402	0.197	4.176	0.041	0.669	0.455	0.984
Treatment after DM	-0.338	0.124	7.392	0.007	0.713	0.559	0.910

**Figure 3:**
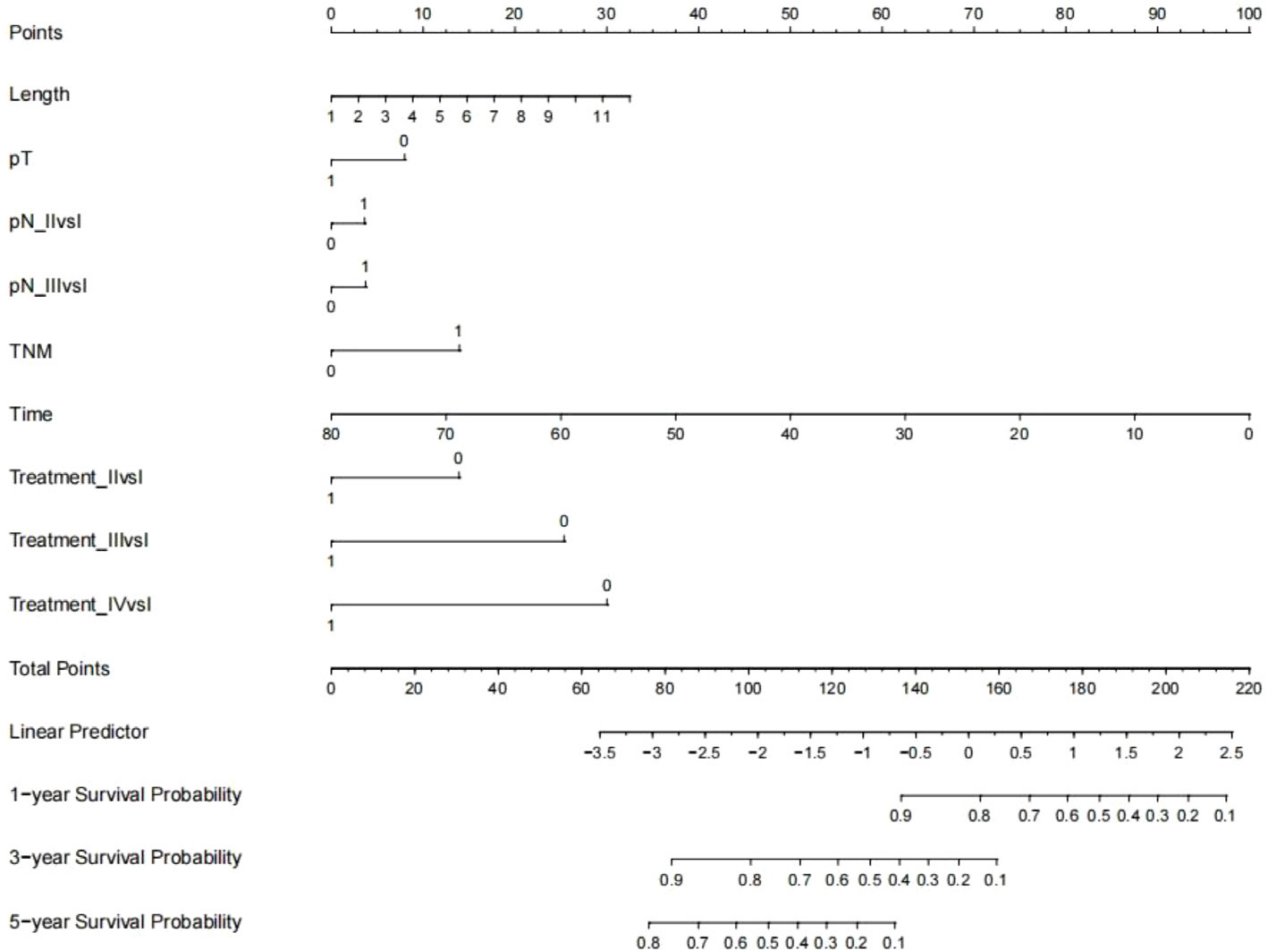
Nomogram of overall survival (Multi-categorical variables such as pN staging and treatment after distant metastasis were transformed into bicategorical variables by setting subvariables).

**Figure 4:**
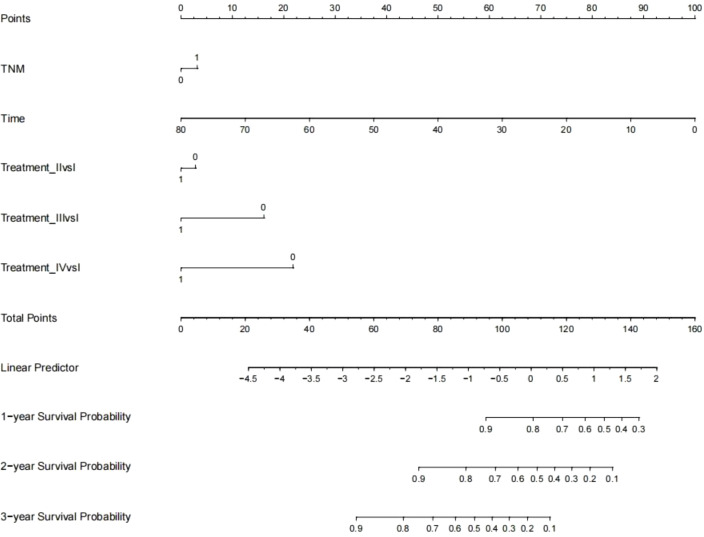
Nomogram of overall survival after oligometastasis (Multi-categorical variables such as treatment after distant metastasis were transformed into bicategorical variables by setting subvariables).

**Figure 5:**
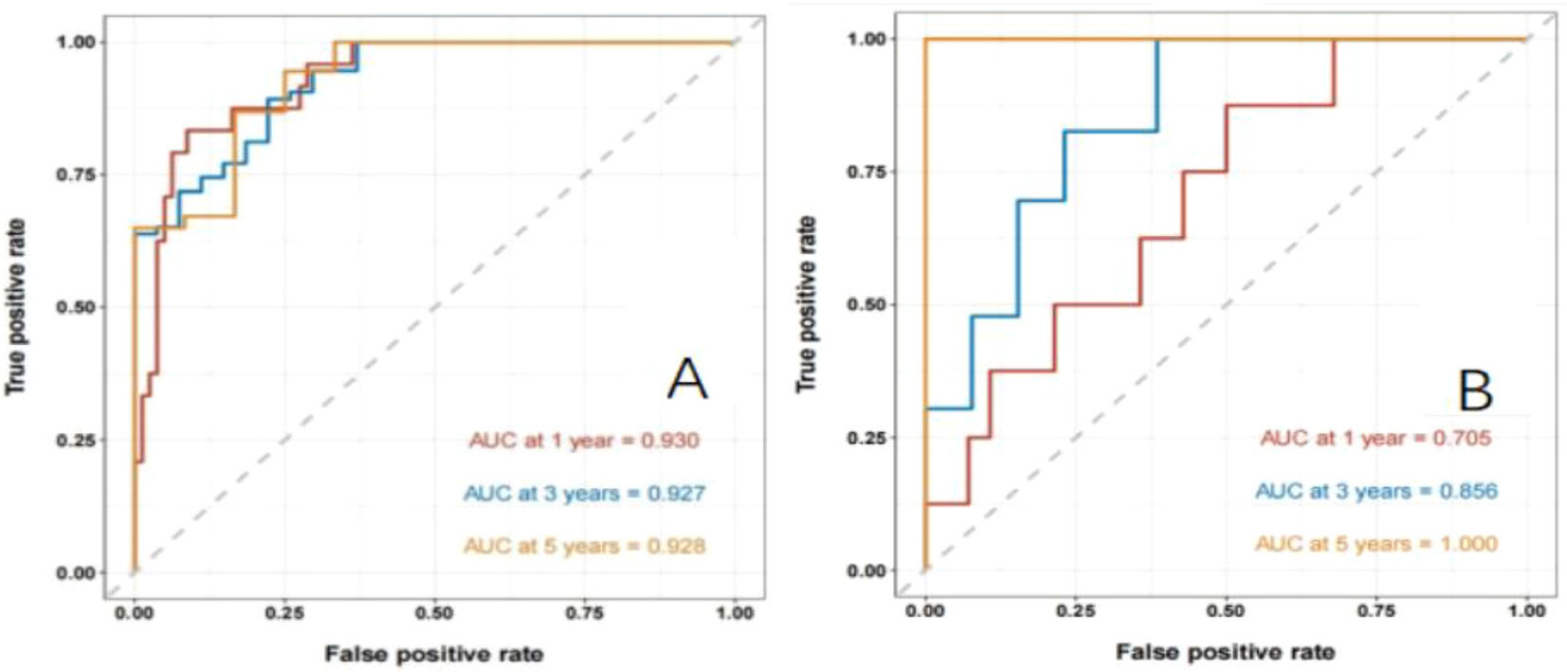
Nomogram to predict the ROC curve of overall OS in patients with postoperative oligometastasis of esophageal cancer (**A**: the training set, **B**: the validation set).

**Figure 6:**
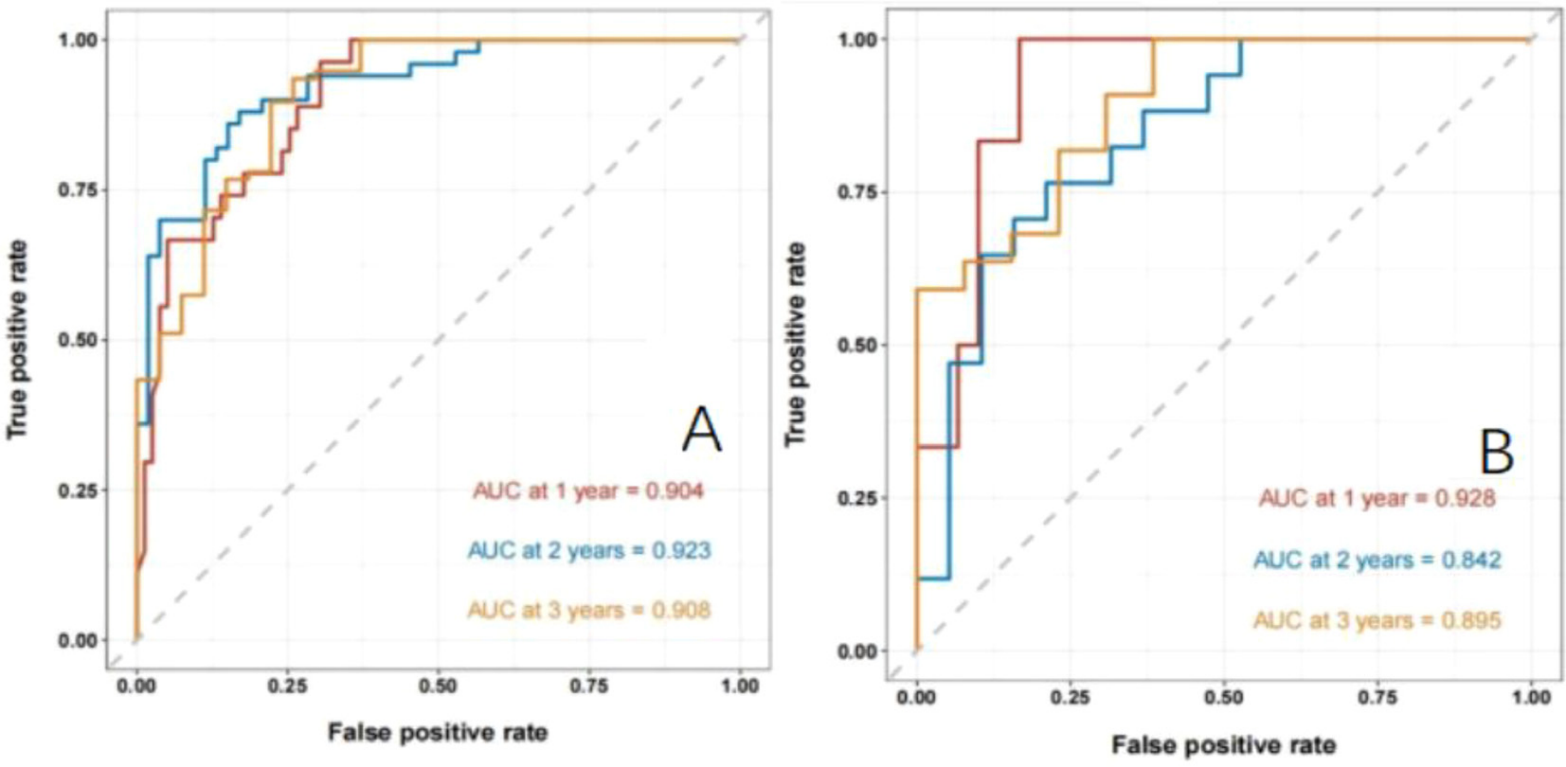
Nomogram model to predict the ROC curve of OM-OS in patients with postoperative oligometastasis of esophageal cancer (**A**: the training set, **B**: the validation set).

**Figure 7:**
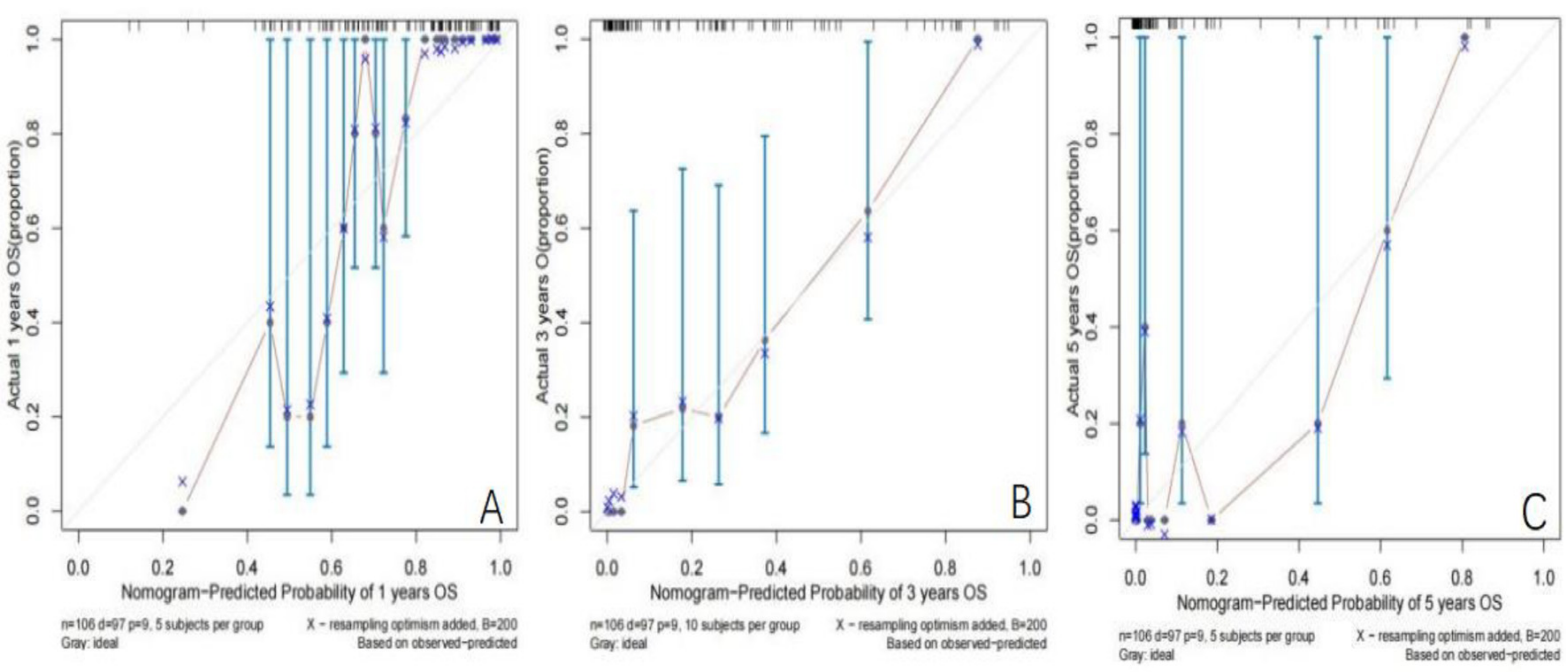
Calibration curve of OS in the training set (**A**: Calibration curve of 1-year OS, **B**: Calibration curve of 3-year OS, **C**: Calibration curve of 5-year OS).

**Figure 8:**
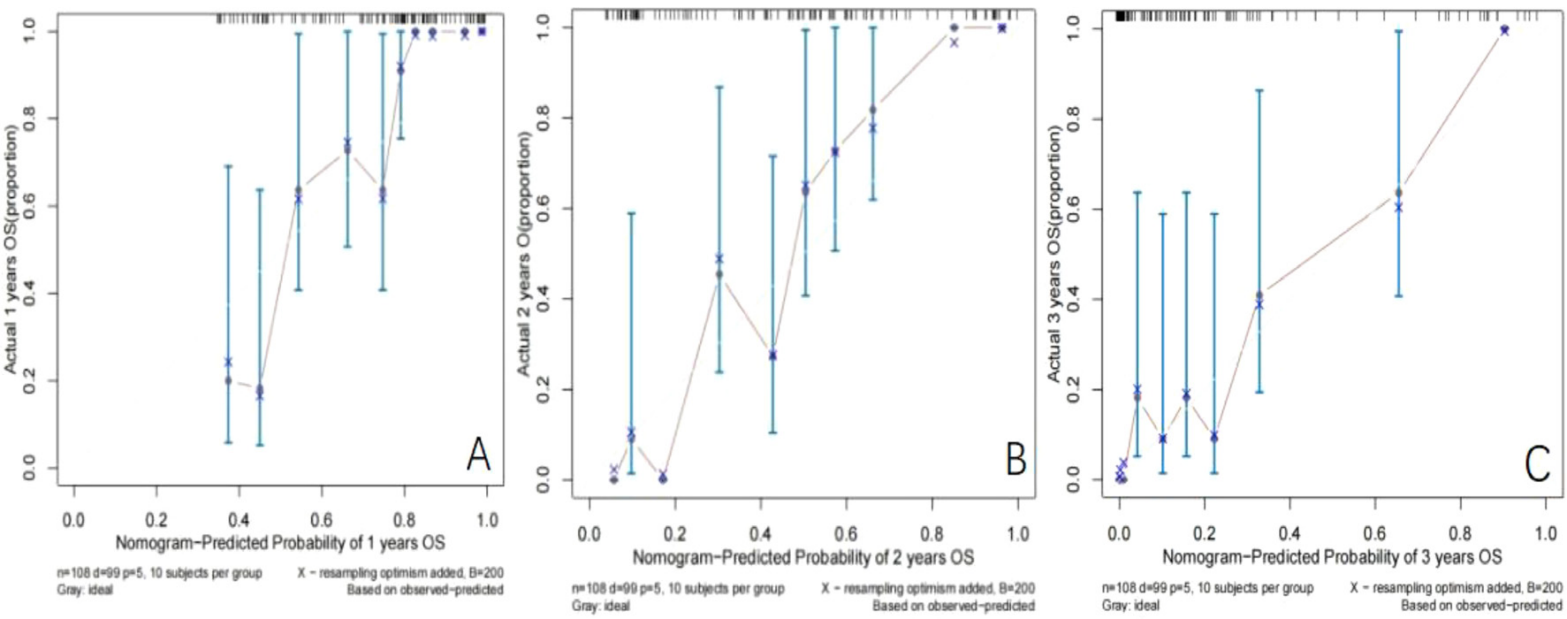
Calibration curve of OM-OS in the training set (**A**: Calibration curve of 1-year OM-OS, **B**: Calibration curve of 2-year OM-OS, **C**: Calibration curve of 3-year OM-OS).

**Figure 9:**
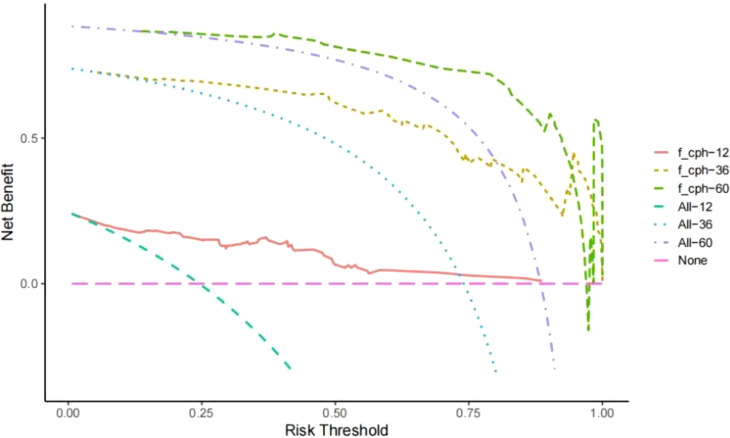
DCA curve of OS in the training set (Solid red lines, dotted brown lines, and dotted green lines indicate the net income of this OS Nomogram model in 1, 3, and 5 years; dotted blue lines, dotted blue lines, and dotted purple lines indicate all deaths in 1, 3, and 5 years; dotted pink lines indicate no deaths).

**Figure 10:**
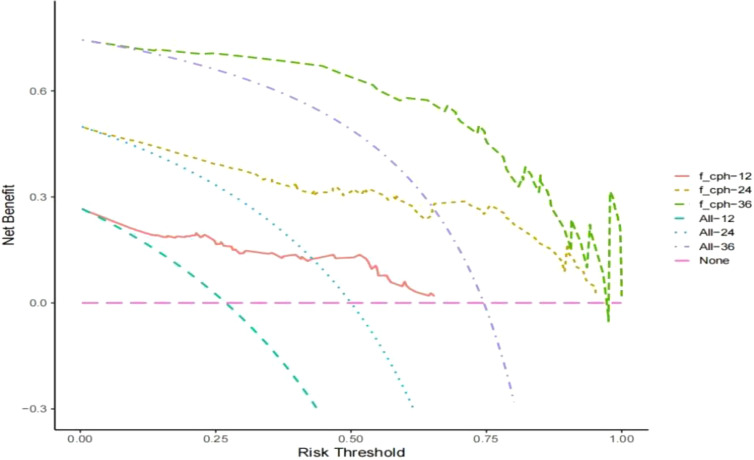
DCA curve of OM-OS in the training set (Solid red lines, dotted brown lines, and dotted green lines indicate the net income of this OM-OS Nomogram model in 1, 3, and 5 years; dotted blue lines, dotted blue lines, and dotted purple lines indicate all deaths in 1, 3, and 5 years; dotted pink lines indicate no deaths).

Establishment and validation of nomogram models for total OS and OM-OS: Prediction nomograms for total OS and OM-OS were established using the Cox multivariate analysis results. The nomogram for total OS was developed using length on X-ray, pT stage, pN stage, TNM stage, TTO, treatment after DM and other factors, and the outcomes were 1-, 3-, and 5-year OS (**Figure 3**). The nomogram for OM-OS was developed using TNM stage, TTO, and treatment after DM, and the outcomes were 1-, 3-, and 5-year OS (**Figure 4**). In the validation of the nomogram models, the AUCs for 1-, 3-, and 5-year total OS (**Figure 5**) were 0.930, 0.927, and 0.928 in the training set and 0.705, 0.856, and 1 in the validation set, respectively; the AUCs for 1-, 2-, and 3-year DM-OS (**Figure 6**) were 0.904, 0.923, and 0.908 in the training set and 0.928, 0.842, and 0.895 in the validation set, respectively. The results indicated that the two models have strong discriminative ability and good clinical promotion and application value. The calibration curves (**Figures 7**, **8**) indicated that the predicted OS and OM-OS were in good agreement with the actual OS and OM-OS. The DCA curves for predicting 1-, 3-, and 5-year total OS and OM-OS are shown in **Figures 9**, **10**, respectively, showing good predictive value and clinical utility for total OS and OM-OS.

### Recurrence patterns

Among the 144 patients included in the study, 102 (70.8%) experienced recurrence or progression during the follow-up period. The lung was the most common site of recurrence, accounting for 40.2% (41/102) of cases, followed by the liver at 22.5% (23/102) and the bone at 17.6% (18/102). Additionally, 19.6% (20/102) of patients experienced recurrence at other sites, including subcutaneous tissue and pleura. The median time to recurrence was 14.2 months, with a range of 1.0–79.3 months. These findings highlight the heterogeneity in recurrence patterns and underscore the need for tailored therapeutic strategies and follow-up protocols based on the site and timing of recurrence (**Table 3**).

**Table 3:** Recurrence patterns analysis.

Recurrence Site	Number of Patients	Percentage (%)	Median OS (Months)
Lung	41	40.2	26.0
Liver	23	22.5	17.0
Bone	18	17.6	16.0
Others	20	19.6	–
Multiple Sites	20	19.6	8.0

## Discussion

Before the immunotherapy era, platinum-based combination chemotherapy was the standard treatment for patients with inoperable metastatic esophageal cancer (15). In the immunotherapy era, immunotherapy or combined chemotherapy has provided a new treatment option for patients with metastatic esophageal cancer (16). However, in the real world, not all patients are suitable for immunotherapy, and even if they receive immunotherapy, the reported results are not satisfactory (17). In addition, in view of strong surgical indications for oligometastatic patients, it is of great clinical significance to explore radiotherapy or chemotherapy as treatment options for metastatic esophageal cancer. Compared with multiple metastases, OMs are less invasive and have limited growth potential; therefore patients with OMs have a better prognosis. However, the prognostic factors for patients with soOMEC are still largely unknown. Therefore, this retrospective analysis was conducted to determine the impacts of different treatments on the prognosis of patients with OMs of esophageal squamous cell carcinoma after surgery and to develop and validate nomograms for predicting postoperative survival and OM-OS in this group of patients. The primary analysis focuses on patients with squamous cell carcinoma (SCC) due to its predominant representation in our cohort (124 of 144 patients). While adenocarcinoma (18 cases) and small cell carcinoma (2 cases) were initially included in the study population, their small sample sizes and distinct biological behaviors could introduce bias. Thus, these subtypes were excluded from detailed analyses to reduce potential heterogeneity in treatment effects and prognostic factors. This decision aligns with our study’s aim to evaluate the prognostic factors specific to SCC.

The role of radical radiotherapy in patients with advanced esophageal cancer has been reported, but most studies have not conducted subgroup analyses of patients with OM (18–20).The 5-year postoperative OS rate for the whole group was 25%, and isolated metastases, systemic chemotherapy followed by supplemental local radiotherapy, and earlier N staging were associated with a better prognosis. Liu et al. (21) conducted a phase II clinical trial of stereotactic body radiation therapy (SBRT) combined with chemotherapy in patients with oligometastatic esophageal squamous cell carcinoma with well-controlled primary lesions and ≤3 metastases; a total of 34 oligometastatic patients with 40 metastatic lesions were enrolled, and the 1- and 2-year progression-free survival (PFS) and OS rates were 55.9%, 33.8%, 76.2%, and 58.0%, respectively. Li et al. (22) analyzed 82 patients with oligometastatic esophageal squamous cell carcinoma and divided them into a radiotherapy (RT) group and a no-radiotherapy (NRT) group based on the treatment after OM; the median OS in the RT group and NRT group was 14 months and 7 months, respectively (P=0.016), and the median OS of the patients in the BED ≥60 Gy and BED <60 Gy groups was 16 months and 10 months, respectively (P=0.033). Based on these findings, Li et al. proposed that the treatment was an independent factor affecting patient prognosis. The above results suggest that radiotherapy, as an important means of local therapy, has potential value in patients with oligometastatic esophageal cancer, a conclusion that is supported by the findings of this study. The retrospective analysis of 144 patients with OMEC showed that radiotherapy was an independent factor affecting patient prognosis.

The results of this study showed that TTO was an independent factor affecting patient prognosis, a finding that is similar to the results of previous studies. Previous studies on patients with OMEC after surgery have highlighted the critical role of time to oligometastasis (TTO) as a key prognostic factor. Shorter TTO has been consistently associated with poorer overall survival, emphasizing its importance in predicting outcomes. Additionally, the pathological stage of the primary tumor has also been identified as an independent factor affecting prognosis in patients with OMEC^23,24^. These findings underscore the significance of early detection and timely management in improving survival outcomes for this patient population. In addition, Li et al. (23) reported that the median OS for patients with times from initial diagnosis to metastasis ≥12 months and <12 months was 15 and 10 months, respectively (P=0.026). We believe that TTO may be related to the initial treatment, an important factor related to the individualized biological behavior of patients, and that a shorter TTO may indicate that patients are less sensitive to the initial treatment. Tumor cells grow actively and rapidly and have high invasiveness; therefore, even after metastasis, the survival rate of patients is lower for those with a shorter TTO. In addition, it has been reported that TTO is an independent factor that affects patient prognosis, a finding that may be related to the different time points when metastatic sites are detected, differences in time to metastasis, and different treatment efficacies for metastases at different sites (24, 25).

It has been widely accepted by clinicians that the postoperative pathological stage of esophageal cancer is the most important factor affecting patient prognosis. In this study, pathological tumor-node-metastasis (pTNM) stage was an independent factor affecting the survival of patients after surgery, but it was not an independent factor affecting the survival of patients after OM, indicating that patients with OM are maintained at the same baseline level; therefore, postoperative pTNM staging has no significant effect on the prognosis of patients with metastases.

To further clarify the accuracy of prognostic factors in this group of patients, nomograms were established to quantitatively predict the OS and OM-OS of patients with OMEC after surgery by incorporating independent prognostic factors. The cross-validation of OS and OM-OS showed that each model has a strong predictive ability. In addition, DCA also demonstrated that these nomograms have good predictive ability. Based on the survival risk assessment using the nomograms, aggressive local and systemic interventions are recommended for patients with OMEC. In addition, the nomograms can be used to assess patient-specific survival. For example, if a patient has received aggressive local therapy but still has a high risk of progression and death, closer monitoring and further intensive treatment will be needed, i.e., immunotherapy or targeted drug therapy can be considered; for patients who have not received local therapy and are at high risk of disease progression, further active local intervention and intensive systemic therapy are recommended. We hope that these predictive nomograms can provide a practical reference for the individualized treatment of patients with OMEC in the near future.

At present, existing clinical trials have shown that a comprehensive treatment model combining systemic treatment and local treatment for patients with oligometastatic disease can control the disease for a long time and even lead to a clinical cure. In recent years, as systemic therapy and local therapy in the new era have become more accurate and efficient, the diagnosis and treatment methods and concepts of oligometastatic cancer have also been rapidly changing, especially the development of targeted therapy, immunotherapy and precision radiotherapy, which are promising advances in the treatment of oligometastasis. However, due to large individual differences among patients with different oligometastases and the fact that the gene mutation characteristics of metastases and primary tumors are often quite heterogeneous, it is particularly important to clearly determine the individualized conditions of each patient. Therefore, the treatment of postoperative OMEC requires not only multidisciplinary and comprehensive antitumor therapy but also more refined and modernized patient stratification. In the future, various novel biomarkers may be used to guide clinical decision-making (26).

Shortcomings of this study: The main limitations of this study are the retrospective nature and small sample size. As a single-center retrospective analysis, hidden sources of bias cannot be excluded, which often results in poor agreement. Additionally, the inclusion of small numbers of non-SCC cases in the initial cohort may introduce heterogeneity. While these cases were excluded from detailed analyses to minimize bias, their presence in the dataset could still influence overall findings. Characteristics of adjuvant treatment regimens, including chemotherapy regimen, number of cycles, radiotherapy regimen, and radiotherapy dose, are inconsistent. Furthermore, the generalization of the results of this study may be limited by the number of patients included. Therefore, prospective clinical trials with a focus on specific histological subtypes are needed to further validate these findings and determine the optimal treatment and regimen for patients with OMEC after surgery.

Although this study identified significant prognostic factors for patients with single-organ oligometastasis of esophageal cancer (soOMEC), its retrospective design introduces inherent biases, such as selection bias from single-center data and potential inconsistencies in medical records. These limitations may affect the completeness and uniformity of data collection and restrict the ability to fully control for confounding variables, thereby limiting causal inferences. Furthermore, the single-center nature of the study may reduce the generalizability of findings due to institutional-specific practices, patient demographics, and treatment protocols. While the nomograms demonstrated strong predictive performance in internal validation, external validation using independent, multicenter datasets is essential to confirm their robustness and broader applicability. Future prospective, multicenter studies with diverse patient populations will be crucial to further validate these results and enhance the reliability of the nomograms. The variability in adjuvant treatments could influence survival outcomes and introduce confounding effects. Due to the relatively small sample size of individual treatment subgroups, detailed stratification and analysis were not feasible. Future studies with larger and more diverse cohorts are warranted to evaluate the prognostic impact of specific treatment modalities In conclusion, the overall prognosis of patients with OMEC after surgery is poor, but some patients with OM can benefit from aggressive local therapy combined with systemic chemotherapy, and for those patients with OM appearing at 1 year after surgery, aggressive radiotherapy or combination chemotherapy is expected to improve the prognosis and prolong OS. The nomograms established in this study are effective clinical tools for predicting the prognosis of patients with soOMEC after surgery and can be of great value in predicting patient prognosis and determining treatment options and can guide the individualized treatment of such patients.

## Data Availability

The raw data supporting the conclusions of this article will be made available by the authors, without undue reservation.
